# Companion Value Co-Creation and Well-Being in Older Adults with Chronic Illness: A Cross-Sectional Dyadic Study in Spain

**DOI:** 10.3390/healthcare14050578

**Published:** 2026-02-25

**Authors:** Leticia Suárez-Álvarez, Ana Belén del Río-Lanza, Ana Suárez-Vázquez

**Affiliations:** Department of Business Administration, University of Oviedo, 33006 Oviedo, Spain; adelrio@uniovi.es (A.B.d.R.-L.); anasv@uniovi.es (A.S.-V.)

**Keywords:** well-being, companion-professional communication, coproduction, value-in-use, older chronic patients

## Abstract

**Background:** Companion participation in medical consultations can influence the well-being of older adults with chronic illness, yet the mechanisms underlying this effect remain unclear. This study aimed (1) to examine how companion-reported value co-creation (coproduction and value-in-use) relates to patient-reported multidimensional well-being (psychological, existential, social, and physical), and (2) to test whether these associations vary according to patient and companion characteristics. **Methods:** A cross-sectional dyadic study of 907 patient-companion pairs (N = 1814) was conducted in Spain prior to the COVID-19 pandemic. Companions completed the adapted Spanish Value Co-creation Scale, while patients completed the McGill Quality of Life Questionnaire-Revised (MQOL-R). Construct validity was confirmed via confirmatory factor analysis, and structural equation modeling tested hypothesized relationships using robust maximum-likelihood estimation. **Results:** The model showed good fit (χ^2^/df = 2.41, CFI = 0.96, RMSEA = 0.041). Companion coproduction was positively associated with patient psychological (β = 0.32), social (β = 0.27), and existential well-being (β = 0.29), but not physical well-being. Value-in-use showed small negative associations (β ≈ −0.10 to −0.15), which may reflect relational strain arising when companions’ involvement is excessive or mismatched with patient needs. Coproduction effects were stronger among patients aged ≤75 years. **Conclusions:** Companion coproduction enhances key dimensions of patient well-being, highlighting its role as a relational resource in clinical practice. Conversely, higher companion value-in-use may signal potential relational strain. These pre-pandemic findings provide a baseline for post-COVID chronic care models that aim to actively involve companions and tailor support according to patient age.

## 1. Introduction

The COVID-19 pandemic profoundly disrupted healthcare systems worldwide, leading to the prioritization of acute care and the cancellation or postponement of non-pharmacological consultations and therapies that are essential for the management of chronic conditions [1,2]. For older adults living with chronic illness, continuity of care—already fragile in many settings—was particularly compromised as healthcare services were reorganized or transitioned to remote modalities [1,2]. At the same time, restrictions on companion participation in medical consultations removed a well-established source of communicative, emotional, and practical support, leaving patients more vulnerable when navigating complex medical information and treatment decisions [3]. Although the data for the present study were collected prior to the pandemic, examining companion involvement before these disruptions provides valuable insights for the design of post-pandemic chronic care models that actively and appropriately integrate companions to support patient well-being.

More generally, chronic illness and its management do not occur in isolation but are embedded within relational contexts that frequently involve informal caregivers. Among older populations, caregivers often accompany patients to medical consultations and play an active role in communication, decision-making, and care coordination. Dyadic research approaches explicitly acknowledge this interdependence by conceptualizing the patient–caregiver pair as a meaningful unit of analysis and integrating both perspectives to better understand shared experiences of illness and care. Qualitative and mixed-methods studies have shown that dyadic analyses enhance understanding of care partnership dynamics, yielding insights that are not captured through individual-level approaches alone [4]. Similarly, quantitative research has demonstrated that caregiver involvement significantly shapes patient self-care behaviors and health outcomes in chronic disease contexts [5].

Even prior to the pandemic, the presence of caregivers or companions during healthcare encounters had been recognized as a key component of patient-centered care for older adults with chronic conditions. Companion participation has been associated with improved recall of medical information, enhanced shared decision-making, greater adherence to treatment recommendations, and improved self-management [6]. In addition, caregiver involvement has been linked to improved psychological well-being and a stronger sense of partnership in care [7]. Recent qualitative evidence further indicates that both patients and informal caregivers value being acknowledged as active participants in medical consultations, reporting greater self-efficacy and enhanced mental well-being when companions are included [8]. Together, these findings underscore the importance of understanding not only whether companions are present during clinical encounters, but also how they participate and contribute.

From a theoretical perspective, companion participation can be situated within the value co-creation paradigm in healthcare, which conceptualizes patients, caregivers, and healthcare professionals as active partners in the joint production of care [9]. Within this framework, coproduction refers to active involvement in care processes such as information exchange, coordination, and shared decision-making, whereas value-in-use captures the personal benefits and meanings derived from these interactions. Integrative models of chronic care further emphasize that family caregivers contribute not only to treatment adherence and clinical decision-making but also to patients’ emotional, social, and existential well-being [10]. Taken together, these perspectives provide a robust conceptual foundation for examining how companion participation during medical consultations may influence multiple dimensions of patient well-being.

Despite growing recognition of the role of companions in chronic care, the mechanisms through which their participation influences patient well-being remain insufficiently understood. Much of the existing literature relies on single informants—either patients or caregivers—and focuses primarily on communication quality or satisfaction outcomes. Few studies employ dyadic designs that simultaneously capture both patient and companion perspectives, and even fewer explicitly examine companion participation through the theoretical lens of value co-creation, including both coproduction and value-in-use. As a result, the evidence base remains fragmented, and it is still unclear which aspects of companion involvement are most beneficial, for whom, and under what circumstances.

Against this background, the present study pursued two objectives. The primary objective was to examine the associations between companion value co-creation during medical consultations—operationalized through coproduction and value-in-use—and the multidimensional well-being of older adults living with chronic illness, including psychological, existential, social, and physical domains. The secondary objective was to explore whether these associations varied according to individual and contextual factors, such as patient age, gender, disease severity, and companion age. By adopting a dyadic, paired-data design, this study aims to contribute to a more nuanced understanding of how companion involvement in clinical encounters shapes patient well-being within chronic care settings.

The remainder of the paper is structured as follows. The next section describes the materials and methods, including the sample, measures, and analytic procedures. This is followed by the presentation of the results. The discussion interprets the findings in relation to existing literature and clinical practice, outlines the study’s limitations, and suggests directions for future research. The paper concludes with final considerations.

## 2. Materials and Methods

This study followed the STROBE (Strengthening the Reporting of Observational Studies in Epidemiology) recommendations for observational research and employed a cross-sectional dyadic design. The completed STROBE checklist is provided as Supplementary Materials (Supplementary Table S1).

The dyad—comprising an older adult with chronic illness and the companion who accompanied them to specialist consultations—was treated as the meaningful unit of analysis in order to capture the interdependence inherent in shared healthcare encounters. Data were collected prior to the COVID-19 pandemic as part of a broader research project examining the role of companions in chronic care.

Two online questionnaires were administered, one to patients and one to companions. Participation was voluntary, all respondents provided electronic informed consent, and the study was conducted in accordance with institutional ethical standards and the Declaration of Helsinki.

### 2.1. Study Design, Setting, and Participants

Participants were recruited with the support of trained student collaborators in outpatient specialist clinics (e.g., cardiology, traumatology, endocrinology, oncology) and through community networks. Collaborators informed potential participants about the study, verified basic eligibility criteria, and distributed the link to the online questionnaires. This convenience sampling strategy is commonly used in dyadic healthcare research and was considered appropriate given the exploratory nature of the study and the practical challenges associated with recruiting patient–companion pairs.

Patients were eligible for inclusion if they were aged ≥65 years, had at least one chronic condition requiring specialist care, had attended at least one specialist consultation in the previous 12 months, and were able to provide informed consent. Patients with a clinical diagnosis of dementia or other major cognitive impairment, or those unable to complete the questionnaire due to severe sensory or functional limitations, were excluded.

Companions were eligible if they were aged ≥18 years, were informal (non-paid) caregivers such as relatives or friends, and had accompanied the patient to at least one specialist visit in the previous 12 months. Professional or paid caregivers were excluded.

**Figure 1:**
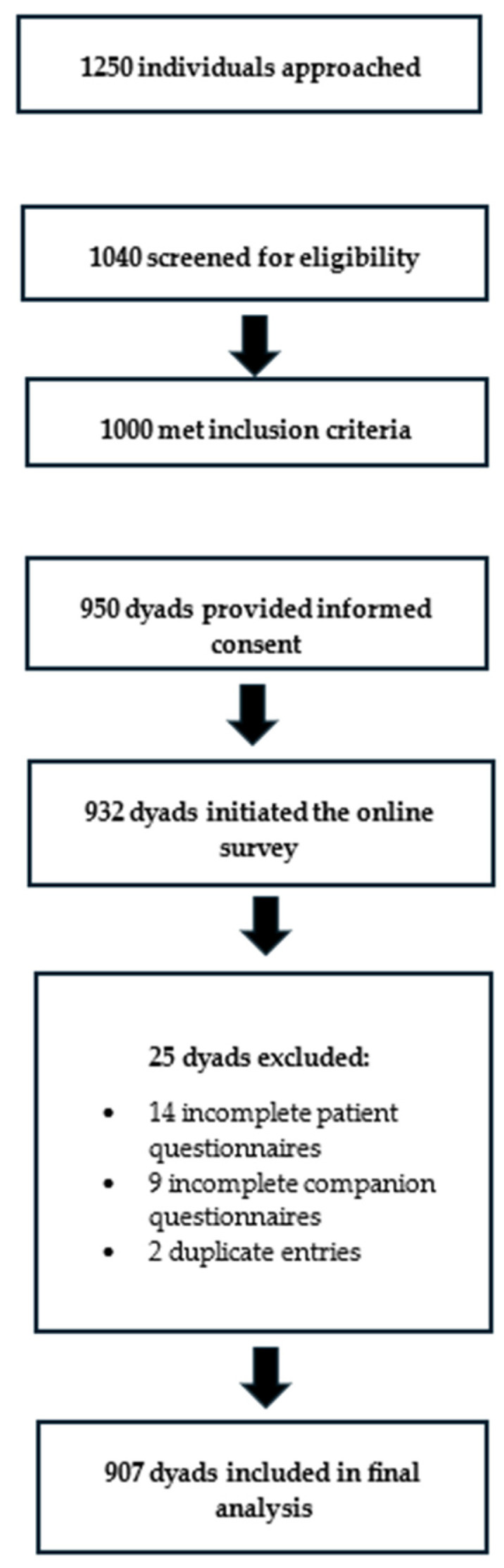
Flow diagram of patient–companion dyads through the study. Note: Counts reflect paired completion. Reasons for non-participation and partial completion were not recorded; this is acknowledged as a potential limitation regarding selection bias.

In total, 1250 individuals were approached; 1040 were screened and 1000 met the inclusion criteria. Of these, 950 dyads consented to participate and 932 dyads initiated the questionnaires. After excluding incomplete or duplicate dyads, the final analytic sample comprised 907 dyads (1814 individuals). Figure 1 illustrates the recruitment process and the number of dyads retained at each stage.

The following sections describe the conceptual foundations used to operationalize the study constructs.

### 2.2. Companion Value Co-Creation: Conceptualization and Measurement

#### 2.2.1. Value Co-Creation: Conceptualization

In organizational research, value co-creation with clients has emerged as a central concept in recent years [11]. According to the Service-Dominant Logic (SDL) proposed by Vargo and Lusch [12], value creation no longer resides solely within organizations but instead emerges through collaborative interactions between service providers and clients. Along similar lines, Prahalad and Ramaswamy [13] argue that all actors involved in an exchange relationship perform a common function by integrating resources and capabilities to generate value.

As emphasized by Ramaswamy and Gouillart [14] and Bird et al. [15], the success of co-creation processes depends on their capacity to enhance the experiences of all parties involved. Empirical research has shown that effective co-creation increases client satisfaction [16,17], strengthens commitment [18], and supports the successful development of new products and services [16,19].

Within the healthcare field, McColl-Kennedy et al. [20] and Lam and Bianchi [21] highlight the importance of patient participation in value creation processes. Similarly, Beirão et al. [22] and Mustak et al. [23] demonstrate that user participation in healthcare value co-creation is associated with improvements in patients’ well-being, health status, and relationships with healthcare professionals.

In the present study, companion value co-creation in healthcare is examined using the framework proposed by Ranjan and Read [24]. This framework was selected because it provides a conceptually robust distinction between coproduction and value-in-use (ViU), capturing both the collaborative processes occurring during service delivery and the experiential value derived from these interactions. This dual structure is consistent with contemporary healthcare research, which conceptualizes value as emerging through resource integration among patients, companions, and professionals and being realized in use during clinical encounters [25]. Empirical studies further indicate that caregivers generate experiential value—such as emotional support, knowledge acquisition, and improved access to care—while also actively participating in procedural and decision-related aspects of treatment [26]. Accordingly, this framework offers a comprehensive lens for examining how companion involvement contributes to multidimensional patient well-being.

Based on an extensive review of the value co-creation literature, Ranjan and Read [24] identified coproduction and ViU as the two fundamental conceptual dimensions of the construct. Each dimension is described below.

##### Coproduction

Coproduction has been defined as client participation in the design and delivery of products or services [27]. Hu and McLoughlin [28] similarly describe coproduction as a process in which service providers “co-work” with clients. Within the SDL perspective, coproduction typically occurs within boundaries established by the service provider, who retains primary control over the process [12]. Building on this view, Ranjan and Read [24] conceptualize coproduction as comprising three dimensions: knowledge exchange, equity, and interaction.

Knowledge exchange plays a central role in identifying current and future needs [29]. Equity refers to the willingness of the provider to share control with consumers, thereby fostering empowerment. Finally, interaction reflects the opportunities for mutual understanding, exchange, and fulfillment of needs that arise through ongoing engagement between actors [30].

##### Value-in-Use (ViU)

Complementing coproduction, the Service-Dominant Logic emphasizes ViU as the value collaboratively generated by clients and service providers through use and experience [12,31]. From this perspective, the client is viewed as an active resource capable of acting and contributing to value creation. Chandler and Vargo [32] later reframed this concept as value in context, emphasizing that value is collectively co-created by multiple actors within specific situational settings.

ViU extends beyond coproduction or exchange and involves individuals actively learning how to use and apply a service in ways that are meaningful within their own context. Consequently, value emerges through the user’s situated experience of use, with individuals assessing and determining the worth of a value proposition based on its practical application [33].

According to Ranjan and Read [24], ViU can be conceptualized along three interrelated dimensions: experience, personalization, and relationship. The experience dimension captures the emotional, empathetic, and memorable aspects that arise through active engagement, generating subjective value [34]. Personalization reflects the extent to which the use process is adapted to individual needs and characteristics, reinforcing the uniqueness of the experience and informing future value exchanges [35]. The relationship dimension refers to the ongoing, interactive communication between users and providers, through which collaboration and commitment enable the co-creation of context-sensitive solutions [36].

While ViU is often conceptualized as a positive outcome of collaborative interactions, recent research highlights its inherently ambivalent nature. Depending on relational dynamics, role expectations, emotional labor, and power asymmetries, ViU may generate not only beneficial experiences but also relational strain, stress, or reduced well-being for one or more actors involved [37,38]. In healthcare contexts—particularly those involving older patients and informal companions—high levels of engagement may therefore produce heterogeneous and sometimes unintended effects.

In this study, the focus is on examining how companion value co-creation relates to patient well-being. The following section describes how this construct was operationalized and measured.

#### 2.2.2. Value Co-Creation: Measurements

Companion coproduction and ViU were measured using items adapted from previously validated scales [27,39,40,41,42,43,44,45,46] and structured in accordance with the conceptual framework proposed by Ranjan and Read [24]. The original instruments have demonstrated adequate reliability and construct validity across organizational and service contexts, supporting their use as a foundation for adaptation (see Appendix A).

Adaptation to the Spanish healthcare context followed internationally recognized guidelines for cross-cultural scale adaptation [47,48,49]. This process included forward translation, back-translation, expert panel review, and cognitive pretesting with 12 older adults and 12 companions to ensure clarity, contextual relevance, and conceptual equivalence of the items.

### 2.3. Patient Well-Being: Conceptualization and Measurement

#### 2.3.1. Patient Well-Being: Conceptualization

Patient well-being represents a central outcome in medical decision-making involving older adults and is widely used as an indicator of the effectiveness and quality of healthcare interventions [50]. In chronic illness contexts, well-being captures not only clinical status but also patients’ subjective experiences of living with disease and treatment over time.

The World Health Organization (WHO) defines quality of life as an individual’s subjective evaluation of their position in life, shaped by cultural context, personal values, goals, expectations, and concerns [51]. In line with Cummins [52], Son et al. [53], and Krok and Gerymski [54], quality of life is closely linked to subjective well-being, understood as the individual’s cognitive and affective evaluation of multiple life domains. This perspective emphasizes personal appraisal processes and integrates both physical and non-physical dimensions of experience.

Building on this tradition, and following Anderson and Ostrom [55] and McColl-Kennedy et al. [20], the present study adopts a multidimensional conceptualization of patient well-being encompassing four interrelated domains: physical, psychological, existential, and social.

Physical well-being refers to functional status and bodily experience and is associated with fatigue, sleep quality, health-related limitations, and self-care activities such as exercise, nutrition, recreation, and rest [20,45]. Psychological well-being reflects emotional and cognitive functioning and aligns with the eudaimonic perspective described by Keyes et al. [56], encompassing anxiety, depressive symptoms, emotional distress, and illness-related cognitive burden [57].

Existential well-being represents a distinct dimension of subjective well-being concerned with meaning in life, purpose, and the individual’s capacity to make sense of illness and adversity [58]. While psychological and existential well-being are conceptually distinct—capturing, respectively, emotional functioning and meaning-making processes—empirical research consistently shows strong associations between them, particularly among individuals living with chronic illness. Studies indicate that meaning-making plays a key role in psychological adjustment to chronic disease, leading to substantial empirical overlap between emotional well-being and existential meaning [59,60]. Similarly, research in existential health psychology highlights that affective functioning and meaning in life are distinct yet interdependent components of well-being, which explains their frequent co-variation in vulnerable populations [61]. This overlap does not indicate conceptual redundancy but rather reflects the dynamic interdependence of emotional and existential processes in contexts of illness.

Finally, social well-being encompasses perceived social support—feeling cared for, valued, and understood—as well as social adaptation, including satisfaction with interpersonal relationships and perceived adequacy of social roles [20,62]. Together, these four domains provide a comprehensive framework for capturing the multidimensional nature of well-being in older adults with chronic conditions.

#### 2.3.2. Patient Well-Being: Measurements

Patient well-being was assessed using the McGill Quality of Life Questionnaire—Revised (MQOL-R), a validated 14-item instrument specifically designed for populations facing chronic and life-limiting illness [63,64]. The MQOL-R was selected because it operationalizes well-being as a multidimensional construct, explicitly including existential meaning while avoiding an exclusive focus on physical symptom burden. This characteristic makes the instrument particularly suitable for older adults with chronic illness, for whom psychological, existential, and social dimensions are central to overall well-being.

Items refer to experiences during the previous month and are rated on a 0–10 response scale (see Appendix A). The MQOL-R has demonstrated good psychometric properties across diverse clinical and cultural contexts, including adequate internal consistency, construct validity, and sensitivity to differences in subjective well-being beyond physical health status [63,64].

### 2.4. Hypotheses Formulation

In healthcare encounters, it is common for vulnerable patients to be accompanied by relatives or friends who provide support during medical visits [65]. Older patients are generally considered particularly vulnerable due to age-related declines in cognitive, biological, and physiological capacities, which are often compounded by the long-term demands of chronic illness [66,67]. This combination of advanced age and chronicity helps explain the high level of family and companion involvement in activities related to the healthcare of older patients, including attendance at medical consultations and participation in care-related decision-making [68,69,70].

A growing body of literature suggests that family and companion involvement in the management of chronic illness contributes to better outcomes for both patients and families. Gilliss et al. [71] argue that family participation enhances illness management and adaptation, while Tamdee et al. [72] emphasize the importance of family involvement in promoting the well-being of older adults. Although these studies support the broader notion that relational and co-creative networks improve patient outcomes, empirical research has rarely focused specifically on the role of the patient’s companion during medical consultations. In particular, little is known about how companions contribute to value co-creation processes within the consultation itself and how these processes relate to patients’ subjective well-being.

Against this background, the present study addresses an important gap in the literature by examining companion value co-creation during medical consultations and its association with older patients’ quality of life, operationalized as multidimensional subjective well-being. In doing so, the study advances current understanding of companion involvement in the care of older patients [73], empirically examines the relevance of value co-creation networks in healthcare services, and responds to calls for further research into the determinants of well-being among older adults with chronic illness [11,74].

Although ViU is often theorized as a mechanism through which service interactions generate positive outcomes, empirical evidence indicates that its effects are not uniformly beneficial. Prior research demonstrates that value co-creation processes may produce heterogeneous outcomes depending on contextual, relational, and emotional conditions. For instance, McColl-Kennedy et al. [75] show that value co-creation activities can also result in value destruction when interactions generate stress, conflict, or misalignment of expectations, roles, and resource contributions among the actors involved. Similarly, Echeverri and Skålén [37,38] demonstrate that co-creation and co-destruction can coexist within the same service encounter. Given this ambivalence, and recognizing that companion participation during consultations may generate positive, neutral, or even negative experiences, it is methodologically appropriate to formulate the hypotheses in a non-directional manner.

Drawing on Ranjan and Read’s [24] conceptualization of coproduction and ViU, the following hypotheses are proposed:

**H1–H4a.**  *Coproduction is associated with physical, psychological, existential, and social well-being.*

**H1–H4b.**  *ViU is associated with physical, psychological, existential, and social well-being.*

In addition to these main associations, prior research indicates that sociodemographic and clinical characteristics may moderate the effects of supportive and co-creative processes on health-related outcomes. Age has frequently been identified as a moderator, with older patients often exhibiting stronger associations between social support and health outcomes due to increased vulnerability and reliance on external resources [76,77]. Gender has also been examined as a moderating factor, as men and women differ in patterns of healthcare utilization and responsiveness to relational support, with women generally reporting greater benefits from social and relational resources [78,79]. Extending this reasoning, the gender of companions may further shape co-creation dynamics, as caregiving roles and relational expectations are often gendered. Finally, disease severity has been shown to condition the strength of psychosocial predictors of well-being, with patients experiencing a higher illness burden deriving greater benefit from supportive involvement [80].

Based on this evidence, the study advances the following moderating hypotheses:

**H5a–H5d.**  *Patient age, patient gender, companion gender, and disease severity moderate these associations.*

**Figure 2:**
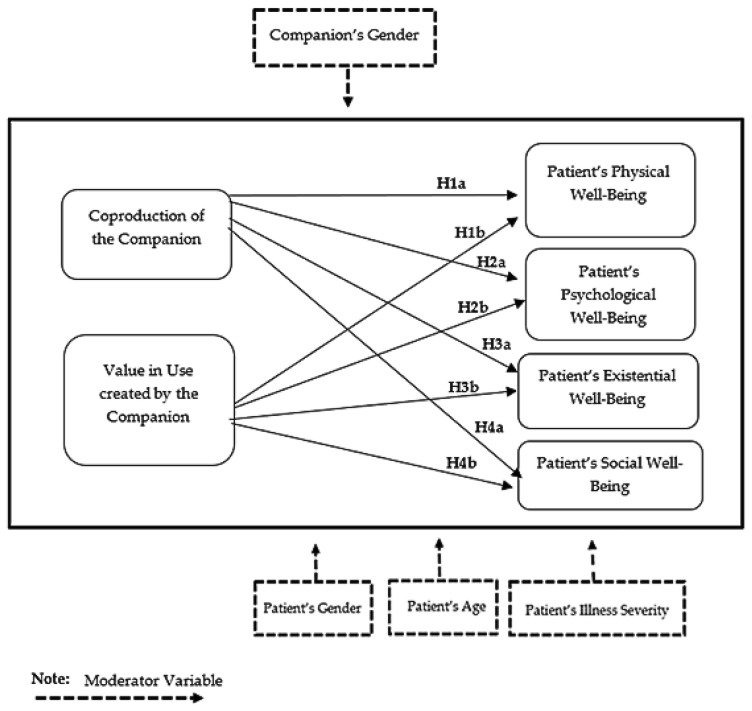
Proposed hypotheses and analysis of moderator variables.

The conceptual model of the study is presented in Figure 2.

### 2.5. Data Collection and Statistical Analysis

Data were collected through two parallel online questionnaires, one completed by patients and the other by their corresponding companions, allowing the preservation of the dyadic structure of the data. The adequacy of the sample size was evaluated following methodological recommendations for structural equation modeling (SEM), which suggest a minimum of 10–15 observations per estimated parameter to ensure model stability and sufficient statistical power. Given the complexity of the proposed dyadic SEM, the number of latent constructs, and the inclusion of moderation analyses, the final sample of 907 patient–companion dyads (N = 1814 individuals) was considered sufficient to reliably estimate the model and detect medium-sized effects.

Missing data were minimal, accounting for less than 2% across all variables. Little’s MCAR test indicated that missingness was completely at random. Considering the dyadic nature of the data and the need to preserve interdependence between patients and companions, listwise deletion was applied, retaining only complete dyads. Under conditions of low and random missingness, this approach is unlikely to introduce meaningful bias and is considered appropriate in dyadic SEM research [81].

EQS Structural Equation Modeling Software (v6.2; Multivariate Software, Inc., Encino, CA, USA) was used to perform the analyses. Robust maximum likelihood estimation was employed to account for potential deviations from multivariate normality, allowing the interpretation of robust standard errors and fit indices [82].

### 2.6. Measurement Validation Procedure

Prior to testing the hypothesized structural relationships, the psychometric properties of all measurement instruments were systematically evaluated. Confirmatory factor analysis (CFA) was conducted to assess the dimensionality, reliability, and validity of the latent constructs. CFA was selected due to the strong theoretical grounding of the measurement models and the use of previously validated instruments adapted to the study context.

Internal consistency reliability was assessed using composite reliability (CR), following the recommendations of Fornell and Larcker [83], with values close to or exceeding 0.80 considered indicative of satisfactory reliability. Convergent validity was examined by evaluating standardized factor loadings and the average variance extracted (AVE), with loadings above 0.50 and AVE values close to or exceeding 0.50 considered acceptable [84]. Discriminant validity was assessed using two complementary criteria: (1) comparison of the square root of the AVE with inter-construct correlations [83], and (2) verification that the confidence intervals of latent factor correlations did not include the value 1 [84].

Model fit was evaluated using multiple indices, in line with contemporary recommendations emphasizing a holistic assessment rather than reliance on single cutoff values [85,86,87].

## 3. Results

### 3.1. Sample Characteristics

The average age of companions was 40 years, and 61% were women. Patients had a mean age of approximately 75 years. The most common chronic conditions were managed within cardiology, followed by orthopedic surgery and traumatology, endocrinology and nutrition, and medical oncology. These characteristics are consistent with the current profile of older adults with chronic illness in Spain, according to data from the Online Aging Laboratory [88].

Given the heterogeneity of chronic conditions represented, an exhaustive list of diagnoses was not feasible. Patients were therefore classified according to the medical specialty responsible for their care, an approach commonly used in research on multimorbidity [89].

### 3.2. Measurement Model Results

The measurement model was first assessed using CFA. To improve model fit while preserving the theoretical meaning of the constructs, several items were removed following standard SEM criteria. The final measurement model comprised two latent constructs related to companion value co-creation—coproduction (F1) and value-in-use (F2)—and four latent dimensions of patient well-being: physical (F3), psychological (F4), existential (F5), and social (F6).

All constructs demonstrated satisfactory internal consistency, with composite reliability values exceeding recommended thresholds (Table 1). Convergent validity was supported by significant standardized factor loadings. Although the AVE for the value-in-use construct was slightly below the conventional threshold (AVE = 0.450), its composite reliability was adequate, and all indicators loaded significantly on the intended factor. As AVE is a conservative estimate of convergent validity, values below 0.50 can be considered acceptable when composite reliability is satisfactory [83]. Given the theoretical relevance of value-in-use within the value co-creation framework [24], the construct was retained to preserve conceptual completeness.

Discriminant validity was confirmed through the Fornell–Larcker criterion and by verifying that confidence intervals for inter-construct correlations did not include unity (Table 2).

### 3.3. Structural Model and Hypothesis Testing

The structural model was estimated using robust maximum likelihood estimation applied to the normalized variance–covariance matrix. Standardized path coefficients and hypothesis testing results are reported in Table 3.

In the full-sample model, companion coproduction (F1) was positively and significantly associated with psychological well-being (β = 0.185, t = 4.748), existential well-being (β = 0.284, t = 6.463), and social well-being (β = 0.332, t = 7.442). The association between coproduction and physical well-being was not statistically significant (β = 0.065, t = 1.429).

Value-in-use (F2) was not significantly associated with physical well-being (β = −0.022, t = −0.469). However, small but statistically significant negative associations were observed with psychological well-being (β = −0.126, t = −2.996), existential well-being (β = −0.102, t = −2.342), and social well-being (β = −0.152, t = −3.577). These coefficients indicate modest effect sizes.

### 3.4. Moderation Analyses

Multisample analyses were conducted to examine potential moderating effects of patient age (≤75 vs. >75 years), patient gender, companion gender, and disease severity. A significant moderation effect emerged only for patient age in the relationship between coproduction and physical well-being. Among patients older than 75 years, coproduction was positively associated with physical well-being (β = 0.412, SE = 0.183, t = 2.253, *p* < 0.05), whereas this association was not significant in the ≤75 group (β = 0.265, SE = 0.187, t = 1.421).

**Figure 3:**
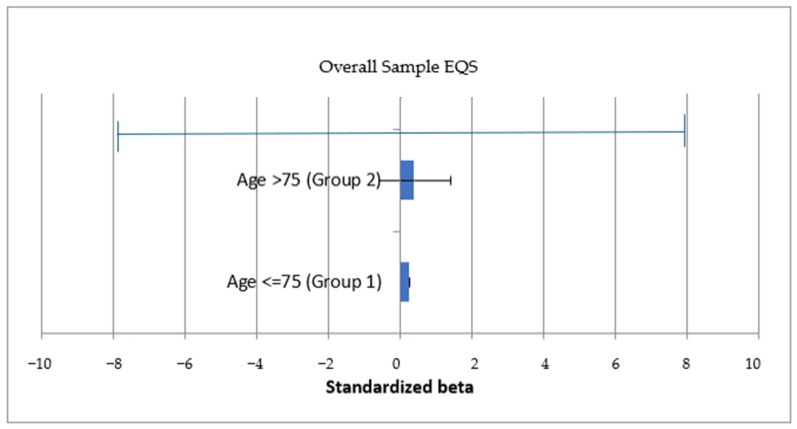
Multisample standardized path coefficients (Coproduction → Physical well-being).

Descriptive t-tests revealed no significant mean differences in physical well-being between age groups (≤75: M = 5.97, SD = 1.65; >75: M = 6.05, SD = 1.71; t(860) = −0.68, *p* = 0.497; Cohen’s d = −0.046). These findings suggest that, despite minimal mean differences, companion coproduction may play a particularly relevant role in physical well-being among older patients (Figure 3).

### 3.5. Model Fit

The Satorra–Bentler scaled chi-square was significant (S-B χ^2^ = 939.94, df = 255, *p* < 0.001), as expected given the large sample size. Fit indices less sensitive to sample size indicated acceptable overall fit: robust RMSEA = 0.054 (90% CI 0.047–0.061), robust CFI = 0.924, and TLI = 0.912. The SRMR value was slightly elevated (0.117), a result commonly observed in complex SEM models with multiple latent constructs and numerous indicators [90,91]. Taken together, these indices support the adequacy of the proposed model, while acknowledging the elevated SRMR as a limitation related to model complexity.

## 4. Discussion

Quality of life in healthcare is closely linked to patients’ subjective well-being and should be examined across its physical, psychological, existential, and social dimensions to capture its multidimensional nature [50]. Understanding how these dimensions are shaped by value co-creation processes is particularly relevant in chronic care contexts, where patients often experience increased vulnerability and reliance on informal support. Despite growing interest in co-creation, empirical evidence on how vulnerable populations actively enhance their well-being remains limited [92]. In this regard, relationship-oriented and person-centred care approaches have been shown to be strongly associated with psychological well-being in older adults, highlighting the importance of participatory and relational processes beyond clinical outcomes [93].

Within this framework, our findings contribute to the emerging literature by demonstrating that companion involvement during medical consultations is differentially associated with distinct dimensions of patient well-being. Consistent with prior reviews of co-creative interventions among older adults [94], companion coproduction was positively associated with psychological, existential, and social well-being. These results reinforce the view that non-physical outcomes are especially sensitive to relational and communicative processes occurring during healthcare encounters. Similar patterns have been observed in community-based and person-centred interventions, where social participation and supportive relationships play a central role in enhancing subjective well-being among older adults [95].

From a service ecosystem perspective, value co-creation integrates resources contributed by multiple actors, including patients, professionals, and informal companions [96]. Companions often help mitigate cognitive, emotional, and informational vulnerabilities faced by older patients, particularly during complex medical interactions. Our results confirm that such coproduction—operationalized as active participation, information exchange, and shared engagement during consultations—supports patients’ sense of psychological security, existential meaning, and social connectedness. These findings align with evidence highlighting the protective role of social networks and informal support in chronic illness management [97,98,99] and extend previous research showing that companions enhance comprehension, perceived safety, and continuity of care [100]. The broader literature further suggests that access to psychosocial resources and social integration is a key determinant of psychological well-being among structurally or socially vulnerable older populations [101].

In contrast, no significant association was observed between coproduction and physical well-being in the overall sample. This distinction underscores the need to interpret physical and non-physical dimensions of well-being separately. Physical well-being is strongly influenced by disease severity, functional status, and clinical trajectories, which were not directly measured in this study. Although proxy indicators related to assistance with appointments, medication management, and logistics were considered, future research should incorporate standardized clinical measures to reduce residual confounding and strengthen causal interpretation.

Interestingly, value-in-use (ViU) exhibited small but statistically significant negative associations with some non-physical well-being dimensions. These effects should be interpreted cautiously, given their modest magnitude. Prior research suggests that ViU may generate both positive and negative outcomes depending on actor perspective, relational dynamics, and information asymmetry [102,103,104]. High levels of companion engagement may sometimes introduce tension, role ambiguity, or misaligned expectations, particularly when patients perceive reduced autonomy or increased dependence [105]. Moreover, this study did not assess companions’ own well-being or burden, despite evidence that caregiving roles can impose emotional and physical strain [106,107]. Empirical evidence from caregiver research further indicates that intensive informal caregiving may be associated with psychosocial distress and relational strain, with gendered patterns in burden and support that can indirectly affect care dynamics [108]. Accordingly, the observed negative coefficients should not be interpreted as clinically meaningful detriments but rather as modest statistical associations reflecting the complexity of relational value creation.

Moderation analyses further revealed that patient age plays a critical role in shaping the relationship between coproduction and physical well-being. While no significant effect emerged at the aggregate level, a positive association was identified among patients older than 75 years. Importantly, this moderation effect reflects differences at the latent level rather than mean-level differences between age groups. SEM-based moderation captures variation in underlying constructs and therefore provides stronger evidence than comparisons based solely on observed means [109,110]. These findings suggest that companion involvement may be particularly relevant for perceived physical functioning among the most vulnerable patients, even when such differences are not evident descriptively.

Age-specific patterns likely reflect differences in functional limitations, cognitive load, and care complexity. Patients over 75 often experience greater challenges related to multimorbidity, treatment adherence, and information processing, making companion support especially salient [76,111]. Consistent with this interpretation, population-based evidence shows that cognitive functioning is positively associated with subjective well-being in older adults, suggesting that increased cognitive vulnerability may heighten the benefits of informational and relational support during healthcare interactions [112]. In contrast, younger-old adults may retain greater autonomy or access alternative support mechanisms, attenuating the observable impact of coproduction on physical outcomes. Across age groups, however, companions consistently contributed to social well-being, highlighting their enduring relational role.

The relevance of these findings is further underscored by the COVID-19 pandemic, during which companion presence was frequently restricted in healthcare settings. Such restrictions disrupted communication, advocacy, and emotional support, disproportionately affecting older and vulnerable patients [113]. Qualitative studies conducted during the pandemic reported heightened distress among relatives due to visitation bans and reduced trust in healthcare systems [114], while survey evidence showed that social support facilitated telehealth uptake and continuity of care among older adults [115]. These experiences emphasize the importance of safeguarding relational support mechanisms during both routine and crisis conditions.

### 4.1. Perspectives for Clinical and Assistive Practice

The findings of this study have several implications for clinical and assistive practice in chronic care. First, the consistent positive associations between companion coproduction and psychological, existential, and social well-being support the systematic integration of companions into medical consultations. Previous research demonstrates that companions enhance communication quality, shared decision-making, and recall of medical information, particularly among older adults with multimorbidity [100,116]. Our results extend this evidence by highlighting broader well-being benefits beyond informational outcomes. These findings are also consistent with person-centred care models that emphasize relational continuity, patient participation, and respect for individual values as core drivers of psychological well-being [117].

Second, the modest negative associations observed for ViU indicate that companion involvement is not uniformly beneficial. Excessive responsibility, relational strain, or poorly managed dynamics may inadvertently undermine patient well-being, consistent with studies linking caregiver burden and over-involvement to reduced interaction quality [118,119]. Clinicians should therefore attend not only to companion presence but also to the quality and balance of the companion–patient relationship.

Third, the moderating role of age underscores the need for age-sensitive communication strategies. As sensory decline, cognitive load, and communication barriers increase with age, tailored approaches that adapt companion involvement to patients’ functional and cognitive profiles may help optimize outcomes [120]. Structured guidance for companions—clarifying roles, expectations, and boundaries—may further enhance their supportive potential.

Finally, as healthcare systems increasingly adopt hybrid in-person and telehealth models, companions may play an expanding role in facilitating communication and patient confidence during remote encounters. Emerging evidence suggests that companion involvement improves engagement and continuity in telemedicine, particularly for older adults with chronic illness [121]. Developing protocols that support effective companion participation in both physical and virtual settings may strengthen post-pandemic care pathways.

### 4.2. Limitations

Several limitations should be acknowledged. The cross-sectional and self-reported design precludes causal inference and may be subject to recall, social desirability, and selection biases. The sample, restricted to older adults with chronic conditions in Spain, limits generalisability to other healthcare systems and cultural contexts. The heterogeneity of conditions necessitated classification by medical specialty, and subgroup analyses by specific diagnoses or dependency levels were not feasible.

Although SEM provided robust estimates of latent relationships, unmeasured confounders remain possible. The study did not include objective clinical indicators such as functional status, comorbidity indices, or clinician-rated severity measures, which limits contextualisation of physical well-being outcomes. Additionally, companions’ well-being, burden, and emotional state were not assessed, constraining interpretation of ViU-related findings.

### 4.3. Future Research

Future research should employ longitudinal and mixed-methods designs to clarify causal pathways linking companion involvement and patient well-being [122]. Incorporating standardized measures of companion burden, stress, and well-being is essential to disentangle reciprocal effects within dyads [123]. Inclusion of clinical covariates—such as validated functional status scales, comorbidity indices, and adherence measures—would further enhance interpretability of physical outcomes.

Comparative studies across cultural and healthcare contexts are also needed to assess the generalisability of these findings. Prespecified subgroup analyses by age, gender, dependency, and chronic condition, combined with both SEM-based estimates and conventional effect sizes, would improve transparency and interpretability [124]. Finally, systematic evaluation of digital and hybrid modalities for companion engagement is warranted, given growing evidence on digital interventions for older adults [125].

## 5. Conclusions

This study advances understanding of value co-creation in healthcare by distinguishing between companion coproduction and value-in-use and examining their differential associations with multiple dimensions of patient well-being. Companion coproduction was consistently associated with improved psychological, existential, and social well-being, while perceived physical well-being benefited primarily among patients over 75 years of age.

These findings reinforce the view that companions are not peripheral actors but integral contributors to chronic care delivery. Evidence from prior studies indicates that informal caregivers improve adherence, comprehension, emotional adjustment, and satisfaction with care, supporting our results [113,126,127]. Systematic integration of companions into care processes—supported by clinician training, structured protocols, and ethical safeguards—can enhance relational quality while preserving patient autonomy [100,128,129]. Broader evidence from person-centred and psychosocial research further supports the role of relational and social resources as key determinants of well-being in later life [93,101].

The COVID-19 pandemic highlighted the consequences of restricting companion participation and underscored the need for resilient, relationship-centred care models capable of adapting to crisis conditions [114,115,130,131,132]. From a policy perspective, recognizing companions as central actors in chronic care can promote equity, reduce pressure on formal services, and improve multidimensional outcomes. Conceptually, this study supports a shift from patient-centred to relationship-centred care, emphasizing collaborative agency among patients, companions, and professionals in shaping meaningful and sustainable care experiences [123,133,134,135,136,137].

## Figures and Tables

**Table 1:** Validation of measurement scales (I).

Concept	Standardized Parameters	Robust t Value	Composite Reliability	AVE
Coproduction (F1)			0.879	0.510
COPRO2	0.664	20.616		
COPRO4	0.748	25.525		
COPRO5	0.732	26.244		
COPRO6	0.685	20.787		
COPRO9	0.775	27.533		
COPRO10	0.703	22.803		
COPRO11	0.683	21.385		
Value-in-use (F2)			0.800	0.450
ViU14	0.576	16.882		
ViU 15	0.756	25.209		
ViU 16	0.652	19.933		
ViU 17	0.728	25.596		
ViU 21	0.613	19.588		
Physical Well-Being (F3)			0.756	0.512
PHYSWB1	0.804	26.985		
PHYSWB2	0.581	14.787		
PHYSWB3	0.743	23.226		
Psychological Well-Being (F4)			0.910	0.720
PSYWB4	0.909	46.210		
PSYWB5	0.860	37.251		
PSYWB6	0.919	46.486		
PSYWB7	0.685	23.911		
Existential Well-Being (F5)			0.774	0.536
EWB8	0.658	18.416		
EWB9	0.707	21.775		
EWB10	0.821	28.151		
Social Well-Being (F6)			0.780	0.547
SWB12	0.883	28.271		
SWB13	0.623	20.787		
SWB14	0.687	17.963		

Note: S-B χ^2^ (260) = 715.1283 (*p* < 0.000) robust-RMSEA = 0.044 (90% CI = 0.038–0.051) SRMR = 0.049 GFI = 0.927 AGFI = 0.908 robust-NFI = 0.923 robust-CFI = 0.949 TLI = 0.932. COPRO = Coproduction; ViU = Value-in-use; PHYSWB = Physical Well-being; PSYWB = Psychological Well-being; EWB = Existential Well-being; SWB = Social Well-being; AVE = Average Variance Extracted.

**Table 2:** Validation of measurement scales (II).

Correlation		Std Error	95% Confidence Interval	
F1–F2	0.656	0.031	0.594	0.718
F1–F3	0.052	0.043	−0.034	0.138
F1–F4	0.118	0.039	0.040	0.196
F1–F5	0.230	0.042	0.146	0.314
F1–F6	0.254	0.042	0.170	0.338
F2–F3	0.014	0.046	−0.078	0.106
F2–F4	−0.021	−0.041	0.061	−0.103
F2–F5	0.059	0.045	−0.031	0.149
F2–F6	0.037	0.043	−0.049	0.123
F3–F4	0.683	0.030	0.623	0.743
F3–F5	0.541	0.040	0.461	0.621
F3–F6	0.259	0.044	0.171	0.347
F4–F5	0.695	0.030	0.635	0.755
F4–F6	0.455	0.037	0.381	0.529
F5–F6	0.727	0.031	0.665	0.789

Note: F1 = Coproduction; F2 = Value-in-use; F3 = Physical Well-being; F4 = Psychological Well-being; F5 = Existential Well-being; F6 = Social Well-being.

**Table 3:** Testing of the proposed conceptual model.

Hypotheses	Standardized Path Coefficient (β)	t-Value	Hypotheses Testing Results
H1a: The coproduction of the companion → the patient’s physical well-being	+0.065	1.429	Not Supported
H1b: The ViU created by the companion → the patient’s physical well-being	−0.022	−0.469	Not Supported
H2a: The coproduction of the companion → the patient’s psychological well-being	+0.185	4.748	Supported
H2b: The ViU created by the companion → the patient’s psychological well-being	−0.126	−2.996	Supported
H3a: The coproduction of the companion → the patient’s existential well-being	+0.284	6.463	Supported
H3b: The ViU created by the companion → the patient’s existential well-being	−0.102	−2.342	Supported
H4a: The coproduction of the companion → the patient’s social well-being	+0.332	7.442	Supported
H4b: The ViU created by the companion → the patient’s social well-being	−0.152	−3.577	Supported
**Moderator variable**		**Multisample analysis**	
Patient’s gender		Non-significant	
Companion’s gender and age		Non-significant	
Age of the patient		Significant effect	

Note: S-B χ^2^ (255) = 939.94, (*p* < 0.001); robust-RMSEA = 0.054 (CI 90%: 0.047–0.061); SRMR = 0.117; GFI = 0.910; AGFI = 0.885; robust-NFI = 0.899; robust-CFI = 0.924; TLI = 0.912.

## Data Availability

The raw data supporting the conclusions of this article will be made available by the authors on request.

## Supplementary Materials

The following supporting information can be downloaded at: https://www.mdpi.com/article/10.3390/healthcare14050578/s1, Table S1: STROBE checklist for cross-sectional studies.

## Appendix A. Measurement Scales Employed

COPRODUCTION: EXCHANGE OF KNOWLEDGE [24,43]
1. *The doctor is receptive to my opinions and suggestions that may help to improve the medical care that my relative needs.* 2. The doctor provides me with sufficient information to understand my relative’s illness. 3. *I would gladly spend time sharing ideas and suggestions with the doctor that could improve the medical care that my relative needs.* 4. The doctor creates a pleasant environment that allows me to present my ideas and suggestions in relation to the illness of my relative.
COPRODUCTION: EQUITY [24,45]
5. The doctor can easily know my preferences with regard to the health care my relative needs. 6. The doctor’s manner of proceeding is in line with what I consider to be correct. 7. *The doctor considers my role as equally as important as his/hers as far as my relative’s health care is concerned.* 8. *The doctor and I jointly decide the final decisions that affect my relative.*
COPRODUCTION: INTERACTION [24,43,46]
9. During the doctor’s appointment I can easily express my requests related to the health care that my relative needs. 10. The doctor tends to give the companions relevant information regarding the illnesses of the relatives they accompany. 11. The doctor permits the participation of the companions during the course of the appointment. 12. *I have taken an active role in my interaction with the doctor during the course of the appointment.*
VALUE-IN-USE: EXPERIENCE [24,41]
13. *I remember perfectly my experience as a companion in the doctor’s appointment.* 14. My own participation during the doctor’s surgery could make my experience as a companion different to that of other companions. 15. The doctor is open to introducing changes to the health care that the patients need following suggestions of the patients’ companions.
VALUE-IN-USE: PERSONALIZATION [24,27,40]
16. The usefulness of the doctor’s appointment depends on the participation of the patient’s companion. 17. During the appointment the doctor adapts to the specific needs of each companion. 18. *Different types of companions tend to show different levels of involvement in the course of the appointment or in the health care of their relatives.* 19. *During the appointment the doctor makes the experience of the companions pleasant, offering them more than just the usual benefits derived from the appointment.*
VALUE-IN-USE: RELATION [24,42,44]
20. *When the doctor encourages the participation of the companions, it improves both the appointment and the health care of the patient.* 21. Nowadays I have a good relationship with the doctor I accompany my relative to. 22. *The companions are very pleased with the doctor.* 23. *The doctor has a good reputation (other companions say positive things about him/her).*
Note: Items in italics were ultimately excluded.

WELL-BEING

In the last month, my physical symptoms (for example, pain, nausea, tiredness, or others):

Were not a problem.	0	1	2	3	4	5	6	7	8	9	10	Were an important problem.

In the last month, I felt:

Very bad physically.	0	1	2	3	4	5	6	7	8	9	10	Very well physically.

In the last month, being physically limited to carry out some activity that I wanted to undertake:

Was not a problem.	0	1	2	3	4	5	6	7	8	9	10	Was an important problem.

In the last month, I was depressed:

Not at all.	0	1	2	3	4	5	6	7	8	9	10	Yes, a lot.

In the last month, I was anxious or worried:

Not at all.	0	1	2	3	4	5	6	7	8	9	10	Yes, a lot.

In the last month, I was sad:

Not at all.	0	1	2	3	4	5	6	7	8	9	10	Yes, a lot.

In the last month, when I thought about the future:

I felt no fear.	0	1	2	3	4	5	6	7	8	9	10	I felt terrified.

In the last month, my life:

Had no meaning (without purpose).	0	1	2	3	4	5	6	7	8	9	10	Was full of meaning (of purpose).

In the last month, I felt that I had my life under control:

Not at all.	0	1	2	3	4	5	6	7	8	9	10	Yes, very much so.

In the last month, I felt happy with myself as a person:

Totally disagree.	0	1	2	3	4	5	6	7	8	9	10	Totally agree

When I think about my whole life and the goals that I have achieved in life, I feel that:

I have achieved nothing.	0	1	2	3	4	5	6	7	8	9	10	I have achieved everything.

In the last month, the communication with the people close to me was:

Very difficult.	0	1	2	3	4	5	6	7	8	9	10	Very easy.

In the last month, I felt that my relations with the people close to me were:

Very comfortable.	0	1	2	3	4	5	6	7	8	9	10	Stressful.

In the last month, I felt supported:

Not at all.	0	1	2	3	4	5	6	7	8	9	10	Yes, totally.
