# Supplementary material for: Companion Value Co-Creation and Well-Being in Older Adults with Chronic Illness: A Cross-Sectional Dyadic Study in Spain
Source: Healthcare (Basel). 2026 Feb 25;14(5):578. doi: 10.3390/healthcare14050578 (PMC12984320; doi:10.3390/healthcare14050578)
Supplement: Supplementary file 1 [file healthcare-14-00578-s001.zip › healthcare-4063906-supplementary.pdf]

Table S1. STROBE Checklist for Cross-Sectional Studies

Companion Value Co-creation and Well-Being in Older Adults with Chronic Illness: A Cross-Sectional Dyadic Study in Spain

ITEM	RECOMMENDATION	
**1a**	Indicate the study design in the title or abstract.	✓
**1b**	Provide an informative and balanced summary of methods and findings.	✓
**2**	Explain the scientific background and rationale.	✓
**3**	State specific objectives and hypotheses.	✓
**4**	Present key elements of the study design early.	✓
**5**	Describe the setting, locations, and relevant dates.	✓
**6a**	Provide eligibility criteria and methods of participant selection.	✓
**6b**	Give matching criteria for matched studies. *Not applicable.*	--
**7**	Define outcomes, exposures, predictors, confounders, and effect modifiers.	✓
**8**	Provide sources of data and methods of assessment.	✓
**9**	Describe efforts to address potential sources of bias.	✓
**10**	Explain how the study size was determined.	✓
**11**	Explain handling of quantitative variables.	✓
**12a**	Describe all statistical methods, including confounding control.	✓
**12b**	Describe methods used to examine subgroups and interactions.	✓
**12c**	Explain how missing data were addressed.	✓
**12d**	Analytical methods considering sampling strategy. *Not applicable.*	--
**12e**	Describe sensitivity analyses. *Not performed.*	--
**13a**	Report numbers of individuals at each stage of the study.	✓
**13b**	Give reasons for non-participation.	✓
**13c**	Use of a flow diagram.	✓
**14a**	Provide characteristics of participants and relevant variables.	✓
**14b**	Indicate number of participants with missing data for each variable.	✓
**15**	Report outcome data or summary measures.	✓

ITEM	RECOMMENDATION	
**16a**	Provide unadjusted and adjusted estimates with precision (e.g., 95% CI).	✔
**16b**	Report category boundaries when continuous variables were categorized. *Not applicable.*	--
**16c**	Translate estimates of relative risk into absolute risk. *Not applicable.*	--
**17**	Report other analyses (subgroups, interactions).	✔
**18**	Summarize key results with reference to objectives.	✔
**19**	Discuss limitations of the study.	✔
**20**	Provide cautious interpretation considering objectives, limitations, and evidence.	✔
**21**	Discuss generalizability (external validity).	✔
**22**	Provide source of funding and role of funders.	✔
